# Using routine programmatic data to measure HIV incidence among pregnant women in Botswana

**DOI:** 10.1186/s12963-022-00287-2

**Published:** 2022-03-04

**Authors:** Katrina F. Ortblad, Shreshth Mawandia, Odirile Bakae, Lenna Tau, Matias Grande, Goabaone Pankie Mogomotsi, Esther Mmatli, Modise Ngombo, Laura Seckel, Renee Heffron, Jillian Pintye, Jenny Ledikwe

**Affiliations:** 1 Public Health Sciences Division, Fred Hutchinson Cancer Research Center, 1100 Fairview Ave N, Seattle, WA 98109 USA; 2 Department of Global Health, University of Washington, Seattle, USA; 3 International Training and Education Center for Health, Gaborone, Botswana; 4 Ministry of Health and Wellness, Gaborone, Botswana; 5 International Training and Education Center for Health, Seattle, USA; 6 Department of Epidemiology, University of Washington, Seattle, USA; 7 School of Nursing, University of Washington, Seattle, USA

**Keywords:** HIV testing, ANC, HIV seroconversion, Pregnancy, PMTCT, Botswana, Women

## Abstract

**Introduction:**

Pregnant women in sub-Saharan Africa have high risk of HIV acquisition, yet approaches for measuring maternal HIV incidence using routine surveillance systems are undefined. We used programmatic data from routine antenatal care (ANC) HIV testing in Botswana to measure real-world HIV incidence during pregnancy.

**Methods:**

From January 2018 to September 2019, the Botswana Ministry of Health and Wellness implemented an HIV testing program at 139 ANC clinics. The program captured information on testers’ age, testing date and result, and antiretroviral treatment (ART) initiation. In our analysis, we excluded individuals who previously tested HIV-positive prior to their first ANC visit. We defined incident HIV infection as testing HIV-positive at an ANC visit after a prior HIV-negative result within ANC.

**Results:**

Overall, 29,570 pregnant women (median age 26 years, IQR 22–31) tested for HIV at ANC clinics: 3% (836) tested HIV-positive at their first recorded ANC visit and 97% tested HIV-negative (28,734). Of those who tested HIV-negative, 28% (7940/28,734) had a repeat HIV test recorded at ANC. The median time to HIV re-testing was 92 days (IQR 70–112). In total, 17 previously undiagnosed HIV infections were detected (HIV incidence 8 per 1000 person-years, 95% CI 0.5–1.3). ART initiation among women newly diagnosed with HIV at ANC (853) was 88% (671/762).

**Conclusions:**

In Botswana, real-world HIV incidence among pregnant women at ANC remains above levels of HIV epidemic control (≤ 1 per 1000 person-years). This study shows how HIV programmatic data can answer timely population-level epidemiological questions and inform ongoing implementation of HIV prevention and treatment programs.

## Introduction

In sub-Saharan Africa, a women’s risk of acquiring HIV increases during pregnancy and the postpartum period [1]. The increased risk of HIV acquisition during this life stage may be attributable to physiological changes that accompany pregnancy—including immune, hormonal, and vaginal microbiome changes [1–3]—as well as behavioral, cultural, and societal factors—including the difficulty to negotiate condom use or male partners’ increased number of sexual contacts during this period [1, 4]. For these reasons, the World Health Organization (WHO) strongly advocates for primary HIV prevention programs targeting pregnant women in HIV high-burden settings—including repeat testing during antenatal care (ANC) and pre-exposure prophylaxis (PrEP)—to minimize the risk of perinatal HIV transmission [5, 6].

Limited data are available related to incident HIV infection during pregnancy in sub-Saharan Africa. Additionally, existing estimates are often based on costly research studies that may not be representative of general populations of pregnant women because of continued observation and access to superior services and counseling, limiting generalizability outside of research settings [7]. A more generalizable approach would be leveraging existing HIV programmatic data to measure HIV incidence during pregnancy. Over the past decades, health information systems for HIV prevention and treatment programs were strengthened to inform ministries of health, multilateral organizations, and donors, subsequently increasing the volume and accessibility of HIV programmatic data, creating opportunities for real-time analyses and decision making [8]. A recent WHO report on health informatics in Botswana highlights the development and implementation of a robust Monitoring and Evaluation and Health Information Systems [9].

Botswana has an adult HIV prevalence of 20% [10] and has achieved high coverage of numerous HIV programs, including universal antiretroviral treatment (ART) and prevention of mother-to-child transmission (PMTCT) [11]. In Botswana, it was estimated in 2018 that 91% of people living with HIV knew their status, 92% of these individuals were linked to care, and > 95% of these individuals were virally suppressed [10]. ANC attendance among pregnant women in Botswana is high, at 97%, and in 2004 Botswana became the first country in Africa to routinize HIV testing at each ANC visit [12]. Botswana is currently rolling out PrEP delivery for individuals with high HIV risk, including women with partners living with HIV, and planning is underway to distribute HIV self-tests to pregnant women to deliver to their sexual partners [13]. As HIV testing programs for pregnant women continue to be scaled up in Botswana, monitoring population-level effectiveness in the reduction of maternal HIV incidence will become increasingly important.

We utilized programmatic data from HIV testing programs at routine ANC clinics in Botswana to measure the frequency of previously undiagnosed HIV infection and HIV incidence among pregnant women. Understanding whether routine health information systems can be used to monitor incident maternal HIV infections and subsequent linkage to ART is important for informing ongoing implementation of HIV prevention programs targeting pregnant women [5, 6, 14].

## Methods

### HIV testing support

Since 2003, the International Training and Education Center for Health (I-TECH) has been working in collaboration with the Botswana Ministry of Health and Wellness (MOHW). In 2015, I-TECH began working with MOHW to strengthen implementation of routine HIV testing through the provision of training, mentoring, and continuous quality improvement activities as well as the provision of human resources at 148 public health facilities, including 139 with ANC services [15]. This support includes collection of routine programmatic data to assess the accountability, transparency, and impact of HIV testing programs as well as data quality assessments. The testing registries include limited demographic information on the individuals testing (age, sex, and citizenship) and details on HIV test performed, including testing date, district, modality (tuberculosis clinic, inpatient, outpatient, ANC clinic, voluntary medical male circumcision, voluntary counseling and testing, and index partner testing), and result. For individuals that test HIV-positive, the registries also indicated if this was a first-time positive result and antiretroviral treatment (ART) was initiated.

### Programmatic data collection

To facilitate monitoring of real-time programmatic implementation, the MOHW (with support from I-TECH) expanded an existing health information system to allow for electronic case-based surveillance of individuals accessing HIV testing services. Site-based HIV testing counselors reported HIV testing data using Open Data Kit electronic forms on handheld devices (tablets). The electronic data forms mirrored variables on paper-based registers for consistency. Data quality checks were inbuilt to increase data reliability and validity. Real-time data were transmitted through a secure mobile network, into the national HIV data warehouse servers. A scaled implementation of the system began in October 2017, with all I-TECH-supported facilities using the system by January 2018.

### Data analysis

For our analyses, we limited our sample to women ≥ 15 years of age who underwent HIV testing through ANC care from January 2018 to September 2019. We excluded individuals who previously self-reported testing HIV-positive prior to their first identified ANC visit, enabling us to measure detection of previously undiagnosed HIV infection. Additionally, we excluded women with an indeterminate or missing HIV test result.

We report the number and proportion of women for whom previously undiagnosed HIV infection was detected at their first observed ANC visit. Among women who tested HIV-negative at their first observed ANC visit and had a repeat HIV test at a subsequent visit, we measured time to re-testing and the frequency of HIV seroconversion during ANC follow-up (defined as testing HIV-positive after a prior HIV-negative result within ANC). We only measured follow-up until the first subsequent ANC follow-up visit. For all women who HIV seroconverted, we assumed that seroconversion occurred midway through their observed follow-up period. We calculated the total number of person-years and observed HIV incidence among women HIV testing at ANC clinics.

To identify potential selection bias in our HIV incidence estimate, we compared the characteristics (age, citizenship, location of first ANC visit—i.e., urban vs. rural, determined by testing district) of women who returned verses those who never returned to ANC clinics for HIV re-testing using Pearson’s chi-squared testing. We also used Pearson’s chi-squared test to compare the characteristics of women who did and did not HIV seroconvert during observation in ANC care. We use bi-variable linear and logistic regression models to measure if age, citizenship, or location of the ANC clinic (i.e., urban vs. rural) were associated with testing HIV-positive or initiating ART at any point during the observation period, including the first ANC visit. We used Stata/SE 16 (College Station, USA) to conduct all analyses and determined significance at the *p* < 0.05 level.

## Results

**Fig. 1:**
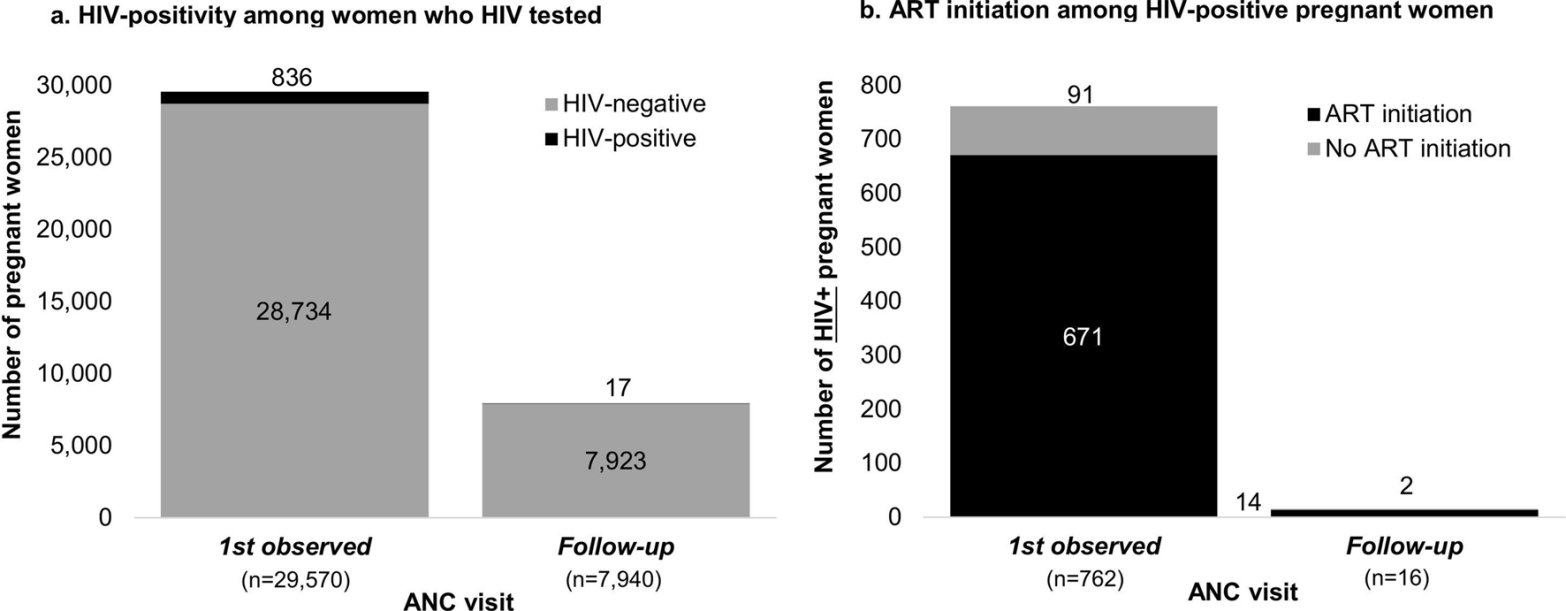
HIV testing outcomes and ART initiation among pregnant women attending ANC clinics in Botswana. At the first observed ANC visit, 343 women were excluded for the following reasons: < 15 years (*n* = 285), missing test result (*n* = 22), and previous HIV-positive diagnosis (*n* = 71). Follow-up was only among women who tested HIV-negative at the first observed visit

**Fig. 2:**
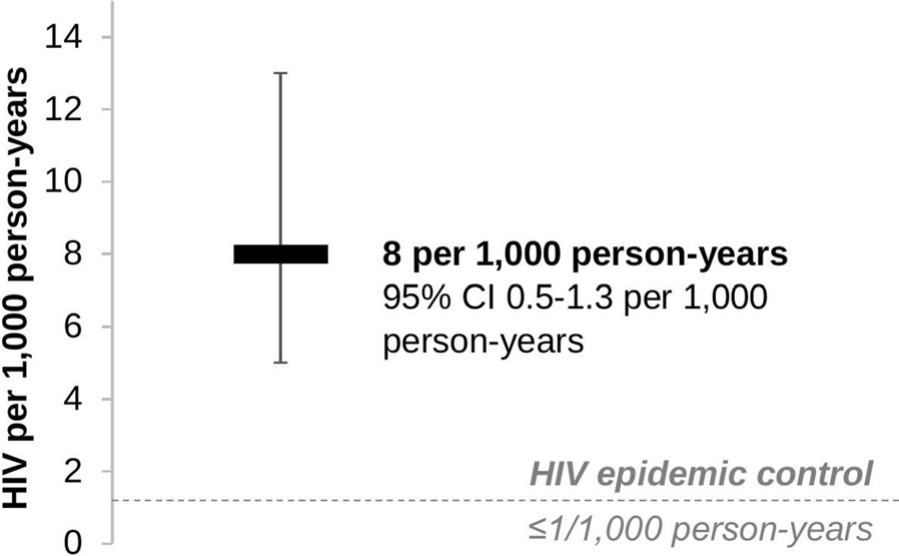
HIV incidence among pregnant women attending ANC clinics in Botswana

From January 2018 to September 2019, 29,570 women tested for HIV at 139 ANC clinics, Fig. 1. The median age of women was 26 years (interquartile range [IQR] 22 to 31 years), roughly half (52%, *n* = 15,516) tested at urban (versus rural) ANC clinics, and the majority were citizens of Botswana (95%, *n* = 28,215). At their first observed visit, 3% of women (*n* = 836) had a previously undiagnosed HIV infection detected and 97% of women (*n* = 28,734) tested HIV-negative. Among the women that tested HIV-negative, 28% (7940/28,734) returned for an ANC follow-up visit at one of the 139 clinics and re-tested for HIV. The median time to HIV re-testing at ANC was 92 days (IQR 72 to 112 days). Overall, 17 previously undiagnosed HIV infections were detected over 2095 person-years of follow-up (HIV incidence rate of 8 per 1000 person-years, 95% confidence interval [CI] 5–13 person-years), Fig. 2. Individuals that HIV seroconverted tended to be younger (median age 23 years, IQR 21 to 27 years), citizens of Botswana (94%, 16/17), who tested in a mix of rural (59%, 10/17) and urban (41%, 7/17) ANC clinics. Among all women who tested HIV-positive at ANC clinics (*n* = 853), 88% (682/775) initiated ART.

When comparing women that did and did not return to the ANC clinics for follow-up and HIV re-testing we found significant differences in age and citizenship, but no significant differences in testing location, Table 1. Compared to women who did not return to ANC clinics, a greater percentage of those who did were ≥ 25 years old (61%, 4712/7732 vs. 58%, 11,709/20,258) and citizens of Botswana (98%, 7760/7940 vs. 95%, 19,701/20,794). The characteristics of those who HIV seroconverted did not significantly differ from those who remained HIV uninfected, Table 1.

**Table 1:** Comparison of characteristics for different groups of pregnant women that HIV tested at 139 ANC clinics in Botswana

Characteristic	Subgroup 1	Subgroup 2	*p-value*^*d*^
Returned an ANC follow-up visit and HIV re-re-tested^a^	Returned and re-tested	Did not return and re-test	
	(*n* = 7940)	(*n* = 20794)	
Age, median (IQR)	26 (22–31)	26 (22–31)	
≥ *25 years old*	4713 (61.0%)	11,709 (57.8%)	< 0.001
Citizen of Botswana	7760 (97.7%)	19,701 (94.7%)	< 0.001
Urban testing location^b^	4207 (53.0%)	10,847 (52.2%)	0.213

HIV seroconverted during observation in ANC care^c^	Did not HIV seroconvert	HIV seroconverted	*p-value*^*d*^
	(*n* = 7928)	(*n* = 17)	
Age, median (IQR)	26 (22–31)	23 (21–27)	
≥ *25 years old*	4919 (62.0%)	7 (41.2%)	0.094
Citizen of Botswana	7744 (97.7%)	16 (94.1%)	0.316
Urban testing location^b^	4400 (53.0%)	7 (41.2%)	0.329

Abbreviations: interquartile range (IQR)

^a^Among women who tested HIV-negative at ANC clinics (*n* = 28,734)

^b^Testing locations categorized into urban and rural testing locations based on testing district

^c^Among women who tested HIV-negative at ANC clinics, returned for an ANC follow-up visit, and re-tested for HIV (*n* = 7940)

^d^*p*-values were measured using Pearson’s chi-squared test; significance was determined at the *p* < 0.05 level

**Fig. 3:**
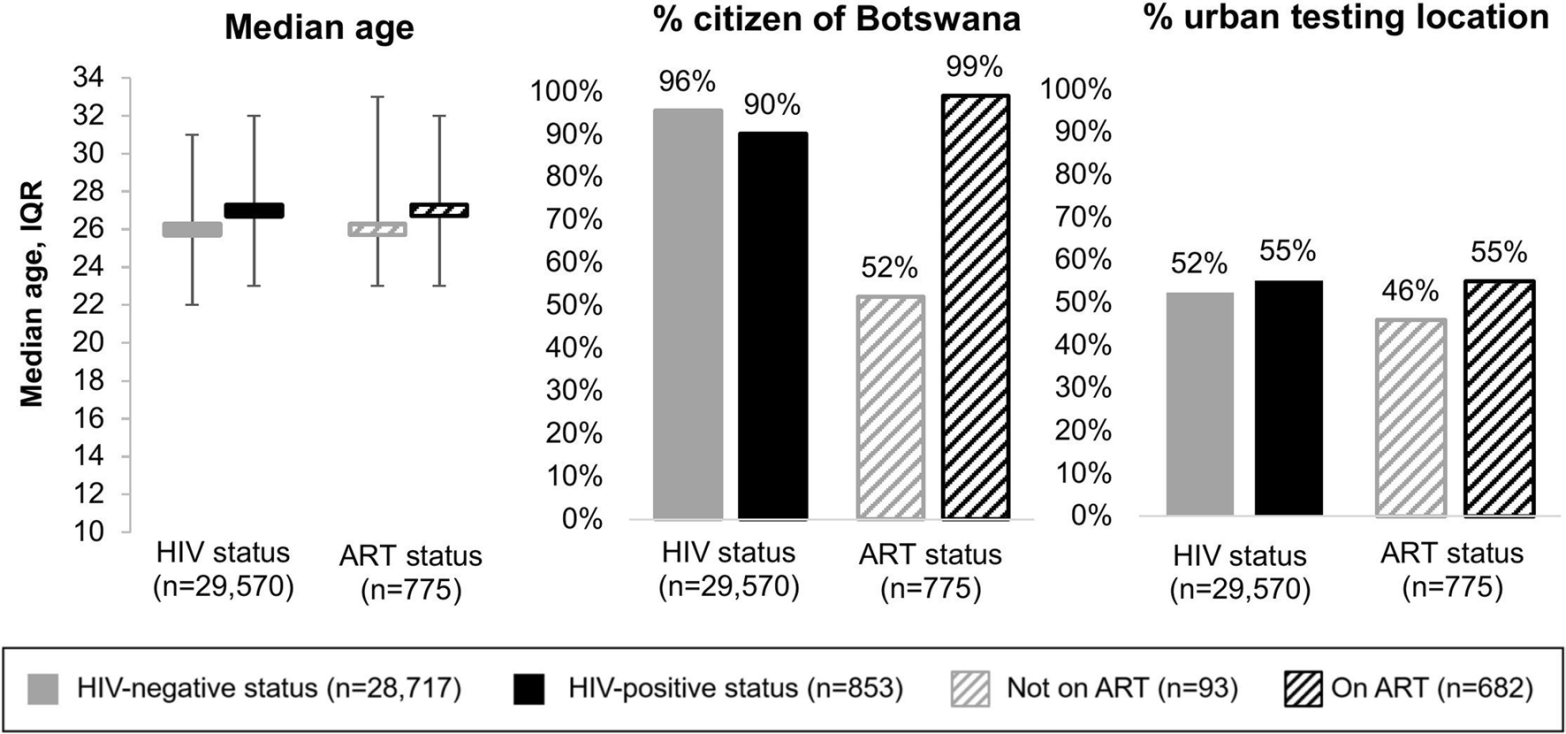
Comparisons in age, citizenship, and ANC clinic location among pregnant women with different HIV diagnoses and ART initiation statuses

We found significant differences in the age and national citizenship of women who tested HIV-negative versus those who tested HIV-positive at ANC clinics during the observation period (including the first ANC visit), and significant differences in national citizenship of women who initiated versus those who did not initiate ART, Fig. 3. Compared to women who tested HIV-negative at ANC clinics, those who tested HIV-positive were older (*p* < 0.001) and a smaller percentage were citizens of Botswana (*p* < 0.001). Compared to women who did not initiate ART, a greater percentage of those who did initiate ART were citizens of Botswana (*p* < 0.001). There were no significant differences in the age of women who did versus those who did not initiate ART (*p* = 0.33). There were also no significant differences in the location of the ANC clinics (e.g., urban versus rural) among women who tested HIV-positive versus HIV-negative (*p* = 0.14) and women who did and did not initiate ART (*p* = 0.13).

## Discussion

The HIV incidence rate measured among pregnant women in this study is lower that measured among pregnant women in other sub-Saharan African settings [7], but similar to other estimates of HIV incidence among members of the Botswana general population [16]. A systematic review of pregnant women in sub-Saharan African settings measured a pooled HIV incidence rate of 47 per 1000 person-years [7]. While this estimate may be higher than that measured in our study (8 per 1000 person-years), the review included sub-Saharan African countries that, relative to Botswana, are often in different HIV epidemic stages [17] and generally have achieved fewer HIV programmatic accomplishments (e.g., levels of HIV testing and treatment coverage) [18]. Meanwhile, the control arm of a large community randomized trial testing the universal test and treat strategy in Botswana—i.e., the Ya Tsie trial or Botswana Combination Prevention Program (BCPP)—measured from 2013 to 2018 an HIV incidence (9 per 1000 person-years) similar to that in our study [16]. Considering the existing levels of population HIV prevalence in Botswana (20%) [10] and the country’s many HIV programmatic accomplishments, the measures of HIV incidence in our study and the Ya Tsie trial are high and remain above levels of HIV epidemic control (≤ 1 per 1000 person-years) [19].

The high levels of HIV incidence among pregnant women, measured using routine programmatic data in this study, suggest that routine HIV testing paired with primary HIV prevention interventions remain critical components of ANC programs in high HIV prevalence settings. Programs for condom distribution [20] and behavioral change interventions [21], initiation of PrEP [22, 23], or secondary distribution of HIV self-tests [14] paired with HIV testing at ANC clinics may improve the lives of pregnant women and reduce perinatal HIV transmission. Additionally, the data from this study suggest that prioritizing older women and women who are not citizens of Botswana for HIV prevention programs might help further improve women’s health and prevent perinatal HIV transmission in Botswana.

This study has a number of strengths. This is the first evaluation to use programmatic data to measure HIV incidence within a HIV high-prevalence setting. Lately, there has been a call to utilize the rich health information systems in low-income, HIV prevalence settings to answer these types of research questions instead of building resource-intensive cohort studies for this purpose [8]. Using programmatic data to inform implementation of HIV prevention efforts is advantageous because the data are readily available (with limited additional costs for analysis) and can be analyzed as programs are ongoing to inform real-time implementation [8].

This study also has weaknesses that are important to note. First, the women who returned to ANC clinics for follow-up and HIV re-testing were significantly different in age and citizenship than those who did not (i.e., they were older and more were citizens of Botswana), which limits the generalizability of our findings. Second, the period of follow-up (i.e., observation in ANC care) was relatively short, as it was limited to the time between when women first tested HIV-negative at a registered ANC visit and when they returned for a subsequent visit, which may limit the reliability of our HIV incidence estimate. Third, limited information was available on the demographics of the women who HIV tested (e.g., education, income) and no details were available on the ANC visit women attended (e.g., their first, second, or third visit), which made it difficult to determine how far along women were in their pregnancy. If our sample captured numerous women at their last ANC visit, we likely underestimated the prevalence of HIV re-testing in this population. Fourth, data were not available for ANC visits at clinics excluded from this assessment. Finally, our sample only included pregnant women who HIV tested at ANC clinics, and thus our estimates of previously undiagnosed HIV infection and HIV incidence may not be generalizable to pregnant women in this setting that do not engage in ANC. However, few women in Botswana do not engage in ANC [24].

## Conclusions

In Botswana, real-world HIV incidence among pregnant women remains above levels of HIV epidemic control (≤ 1 per 1000 person-years) [19]. This study demonstrates that programmatic data from HIV implementation programs can be used to answer timely population-level epidemiological questions (e.g., HIV incidence among pregnant women), which can inform ongoing implementation of HIV prevention and treatment programs. Detection, especially early detection, of HIV among pregnant women and subsequent linkage to ART is important for both the long-term health of the mother [25] and prevention of perinatal HIV transmission [26]. High ANC attendance in Botswana [24] creates the unique opportunity to link women who test HIV-negative (the vast majority) to HIV prevention interventions, such as PrEP [22, 23] or secondary partner distribution of HIV self-tests [14], to maintain their HIV-negative status. To reach elimination of mother-to-child transmission, governments in high HIV prevalence settings should consider analyzing existing programmatic data to inform the real-time implementation of HIV prevention and treatment programs. The findings from this study suggest that continued investment in routine HIV testing paired with other HIV prevention interventions during ANC—a unique period in women’s lives when they are at increased contact with the health system and increased risk of HIV infection [1]—may help reduce HIV incidence and perinatal HIV transmission in Botswana and other similar settings.

## Data Availability

The datasets generated and/or analyzed during the current study are not publically available because of a data agreement between I-TECH and the Botswana Ministry of Health and Wellness, but are available from the corresponding author on reasonable request.

## Abbreviations

ANC: Antenatal care
ART: Antiretroviral treatment
BCPP: Botswana Combination Prevention Program
CI: Confidence interval
HIV: Human immunodeficiency virus
HRDC: Human Resource Development Council
HRSA: Health Resources and Services Administration
I-TECH: International Training and Education Center for Health
IRQ: Interquartile range
MOHW: Ministry of Health and Wellness
PEPFAR: Presidents Emergency Plan for AIDS Relief
PMTCT: Prevention of mother-to-child transmission
PrEP: Pre-exposure prophylaxis
WHO: World Health Organization
